# Modeling a synthetic aptamer-based riboswitch biosensor sensitive to low hexahydro-1,3,5-trinitro-1,3,5-triazine (RDX) concentrations

**DOI:** 10.1371/journal.pone.0241664

**Published:** 2020-11-30

**Authors:** Michael L. Mayo, Jed O. Eberly, Fiona H. Crocker, Karl J. Indest

**Affiliations:** 1 Environmental Laboratory, US Army Engineer Research and Development Center, Vicksburg, MS, United States of America; 2 Central Agricultural Research Center, Montana State University, Moccasin, MT, United States of America; The University of Alabama, UNITED STATES

## Abstract

RNA aptamers are relatively short nucleic acid sequences that bind targets with high affinity, and when combined with a riboswitch that initiates translation of a fluorescent reporter protein, can be used as a biosensor for chemical detection in various types of media. These processes span target binding at the molecular scale to fluorescence detection at the macroscale, which involves a number of intermediate rate-limiting physical (e.g., molecular conformation change) and biochemical changes (e.g., reaction velocity), which together complicate assay design. Here we describe a mathematical model developed to aid environmental detection of hexahydro-1,3,5-trinitro-1,3,5-triazine (RDX) using the DsRed fluorescent reporter protein, but is general enough to potentially predict fluorescence from a broad range of water-soluble chemicals given the values of just a few kinetic rate constants as input. If we expose a riboswitch test population of *Escherichia coli* bacteria to a chemical dissolved in media, then the model predicts an empirically distinct, power-law relationship between the exposure concentration and the elapsed time of exposure. This relationship can be used to deduce an exposure time that meets or exceeds the optical threshold of a fluorescence detection device and inform new biosensor designs.

## Introduction

The machinery of cellular biology excels at reliably sensing weak chemical signals, which are more difficult for traditional electronic detectors to measure because of the low signal-to-noise ratio (SNR). Perhaps unsurprisingly, a long-standing goal of synthetic and molecular biology has been to leverage living matter to improve the fidelity of this low SNR regime. RNA aptamers are a class of promising candidates for this sensing challenge, which are relatively short nucleic acid sequences that typically consist of 15-75 bases flanked by conserved primer binding sites [1]. They bind to targets with high specificity through a combination of their 3D structure, electrostatic interactions, and stacking interactions between aromatic moieties [2]. In addition, they have been used before to bind proteins, metals, antibiotics, dyes, fluorophores, hormones, mycotoxins, and many other small organic molecules [3–6]. Aptamers have several advantages over protein and antibody-based biosensors: they are less complex, and therefore easier and cheaper to synthesize; are more stable across varied thermal and pH environments; possess a more versatile chemistry; display lower immunogenicity; and often exhibit a higher affinity and specificity to the target [1, 5, 7].

One challenge facing aptamer-based sensors is to effectively translate the target-aptamer binding interaction into a detectable signal. This need has led to the development of a variety of signal transducing platforms including biological, electrochemical, and optical sensors [6, 8, 9]. Magnifying such a signal while also maintaining the sensitivity and dynamic range inherent to the aptamer remains a challenging problem. One approach for macroscopic detection of an aptamer-target molecular binding event is to leverage a riboswitch, which consists of an aptamer domain that is capable of interacting with a target, in addition to a regulatory domain that controls expression of an associated gene [10–12]. Substrate binding to the aptamer domain causes a conformational change to the structure of the RNA, which, in turn, controls the termination of transcription, the initiation of translation, or some other alternative splicing regulation [12, 13]. Over 30 classes of riboswitches, representing a wide variety of regulatory mechanisms, have been found to date spanning all three kingdoms of life [14].

Although naturally occurring riboswitches are of interest due to their regulatory roles in cells, they are of limited benefit to applications such as “smart” nanoscale sensor technologies or synthetic genetic circuits [15, 16], which demand a flexible sensing platform that can be tuned toward chemicals or materials of interest. Therefore, a better mechanistic understanding between riboswitch form and function is needed for them to be more widely employed toward an engineering purpose. We assume that kinetics of the riboswitch response are generally rate-limited by the folded conformation of the bound aptamer due to rapid folding/unfolding kinetics [17]; resolving the details of this structural change is one of the most significant challenges to understanding riboswitch function [14]. To accurately predict these functions requires a model that equally describes the statistical equilibrium of stable riboswitch conformations across the free energy landscape, in addition to the non-equilibrium processes that regulate transport of the signal into the cell, aptamer-ligand interactions, and the rates of transcription and translation of the signal [15].

Computational approaches to predicting riboswitch function have focused on identifying riboswitch genes, modeling the folding landscape, and predicting conformational changes [13, 14], and other efforts have leveraged in vitro systems to illuminate riboswitch dynamics at the level of single molecules [12]. In contrast, fewer investigations have focused on modeling in vivo riboswitch mediated signaling in response to ligand binding, wherein complexity in the multiscale dynamics of ligand transport in the cell, aptamer-ligand binding, kinetics of transcription and translation, and the metabolic state of the cell is nontrivial. We aim to help fill this capability gap by developing a holistic modeling framework that is flexible enough to describe the out-of-equilibrium processes needed to translate a bound aptamer state into an observable fluorescent response indicative of activated riboswitch function. Although our model is developed for a specific test chemical (hexahydro-1,3,5-trinitro-1,3,5-triazine), we expect that it can be rapidly “reparameterized” to describe riboswitch activity for a broad set of chemicals and aptamers.

## Materials and methods

### DsRed expression dataset from bacterial culture

We previously described detection of hexahydro-1,3,5-trinitro-1,3,5-triazine (RDX) with *Escherichia coli* (*E. coli*) containing a novel RNA riboswitch [18] developed for an aqueous environment by means of expression of the fluorescent DsRed protein [19]. The expression system consisted of an aptamer domain, linker sequence, and the DsRed Express2 gene. Assays were performed in 48-well plates with 1 ml of LB media and 50 ng/ml ampicillin. A culture of *Escherichia coli* was grown overnight in LB medium supplemented with 50 ng/ml ampicillin. This culture was used to inoculate 48-well plates with an initial optical density at 600 nm (OD_600_) of 0.05. Expression was induced by addition of 0, 0.44, 4.4, and 44 *μ*M RDX followed by incubation at 37 C with constant shaking at 150 rpm for 24 hours. Fluorescence was measured every 10 minutes using a BioTek Synergy HT plate reader (BioTek Instruments, Inc., Winooski, VT) with an excitation wavelength of 530 nm and an emission wavelength of 590 nm. Fluorescence expression data was modeled (see below), and this model was then used to characterize the RDX detection efficacy of the riboswitch, and to provide insight into riboswitch characteristics and assay design constraints for efficient RDX detection. We generated a standard curve using purified DsRed protein (BioVision, Milpitas, CA), and measured fluorescence of the DsRed protein using a BioTek Synergy HT plate reader (BioTek Instruments, Inc., Winooski, VT) as previously described [18], with an excitation wavelength of 530 nm, an emission wavelength of 590 nm, and a PMT sensitivity rating of 35. These data establish a quantitative relationship between protein concentration and fluorescence (S1 Fig).

### Mathematical model of riboswitch activation and response

**Fig 1:**
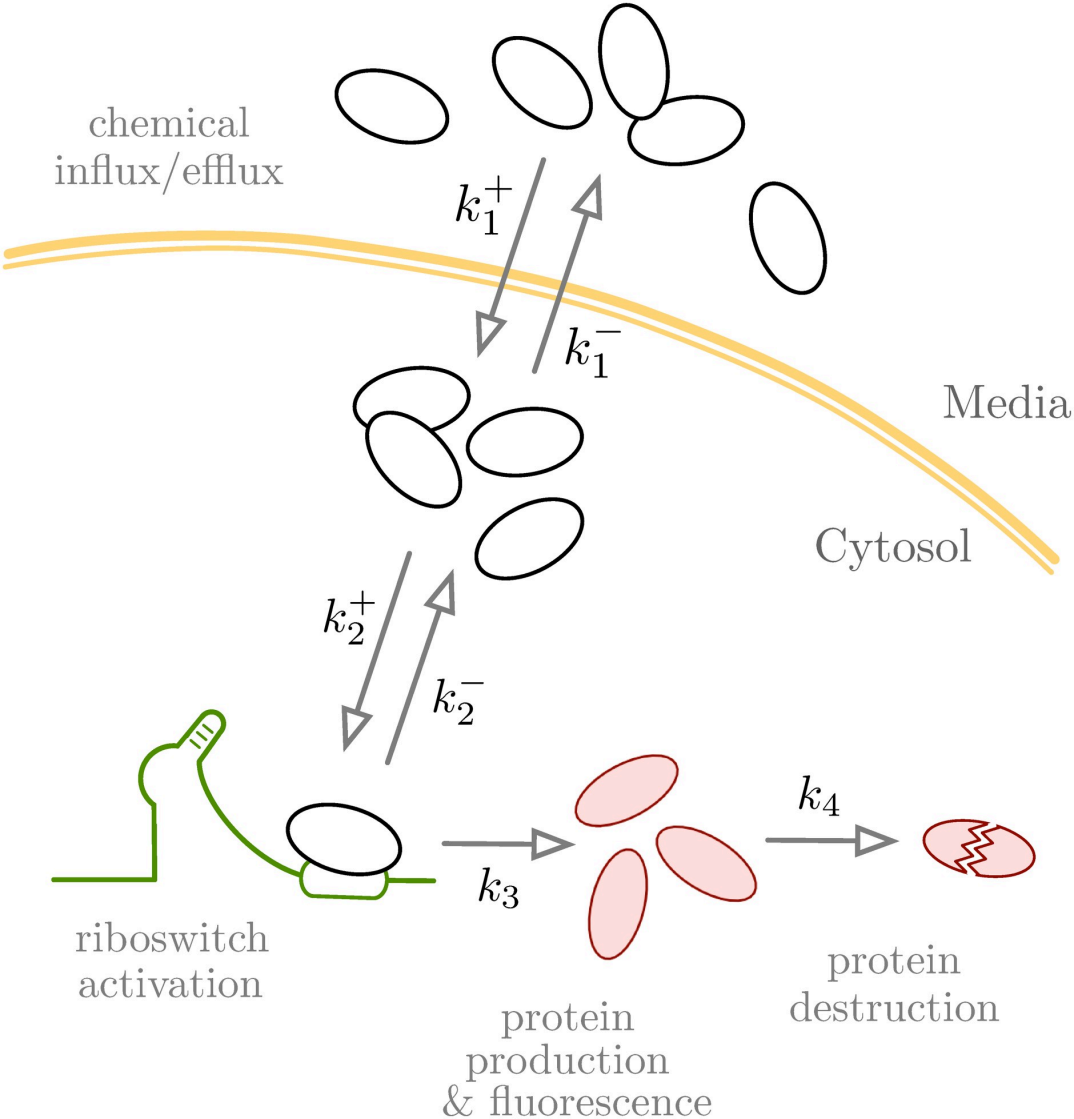
Conceptual model for the aptamer based riboswitch reporter system, as described in Eberly *et al.* [18]. Here, a chemical of interest accumulates within *E. coli* bacterial cells where it binds to the RNA aptamer, initiating translation of DsRed fluorescent protein via the riboswitch system.

At time *t* = 0, we add an aliquot of RDX to a culture of *E. coli* bacteria in the exponential growth phase, and refer to the period of elapsed time, *t*, as the exposure time. Influx mechanisms permit RDX transport across the lipid membranes and into intracellular cytosol, after which we considered only three options for the fate of each molecule: it either binds to an RNA aptamer, is removed from the cytosol via efflux mechanisms, or freely diffuses in an unbound state. Binding of RDX to the aptamer results in a conformational change that permits ribosome binding to the mRNA, which leads to expression of the DsRed gene from which DsRed protein production and degradation follow. The accumulation of DsRed is quantified in terms of a bulk measurement made across the bacterial population via fluorescence imaging, which, according to the DsRed standard curve (S1 Fig), can be directly related to DsRed protein concentrations. Fig 1 illustrates our abstraction of the riboswitch sensing process.

Several assumptions are made about the aptamer-riboswitch-DsRed expression system that reduces its overall complexity and makes it mathematically tractable, but at a cost of deemphasizing the influence of statistical fluctuations that might be important in some experiments with fewer replicates. First, we assume that fluorescence of the DsRed expression system can be explained by using a reaction-limited chemical kinetics formalism applied to biochemical processes within bacterial cells. The implications of this assumption are that correlations between biochemical fluxes extend to at least the size of the cell (i.e., the “well-stirred” hypothesis); reversible transport of RDX across the lipid membranes rapidly establishes a chemical equilibrium quantified by a partitioning constant; aptamer conformation time-scales are short enough that folding dynamics can be neglected in favor of only the presence or absence of the folded state; and that yield of the DsRed protein can be described entirely in terms of the activated riboswitch concentration.

Our second primary assumption is that mean total riboswitch concentrations, [*r*]_tot_, and mean total RDX concentrations, [*c*]_tot_, are fixed, and that these molecules do not degrade significantly over the course of the experiments. In particular, we split the mean riboswitch concentrations between those activated through aptamer binding, [r*](*t*), and those describing aptamers not bound with RDX, [*r*](*t*): [*r*]_tot_ = [r*](*t*) + [*r*](*t*). Mean RDX concentrations can be similarly described, except that we make the distinction between mean RDX concentrations in media outside of the cell, [*c*_out_](*t*), from those that are inside of the cell, [*c*_in_](*t*): [*c*]_tot_ = [*c*_out_](*t*) + [*c*_in_](*t*) + [r*](*t*). Taken together, these assumptions lead to the ordinary differential equations shown in Table 1.

**Table 1:** Rate equations and parameter values for the aptamer-based riboswitch sensor model.

**Model Equations**				
Symbol	Description	Rate Equation		
[*c*_out_]	Mean free chemical concentration in aqueous media	$\frac{d}{dt}[c_{\text{out}}]=k_{1}^{-}[c_{\text{in}}]-k_{1}^{+}[c_{\text{out}}]$		
[*c*_in_]	Mean free chemical concentration within cytosol of bacterial cells	$\frac{d}{dt}[c_{\text{in}}]=k_{1}^{+}[c_{\text{out}}]-k_{1}^{-}[c_{\text{in}}]$		
[r*]	Mean chemical-riboswitch complex within cytosol of bacterial cells	$\frac{d}{dt}[r^{*}]=k_{2}^{+}[c_{\text{in}}][r]-k_{2}^{-}[r^{*}]$		
[*r*]	Mean free riboswitch concentration within cytosol of bacterial cells	$\frac{d}{dt}[r]=k_{2}^{-}[r^{*}]-k_{2}^{+}[c_{\text{in}}][r]$		
[*p*]	Mean DsRed peporter protein concentration within cytosol of bacterial cells	$\frac{d}{dt}[p]=k_{5}+k_{3}[r^{*}]-k_{4}[p]$		
**Model Parameters**				
Symbol	Description	Value		Unit
		Computational	Mathematical	
$k_{1}^{+}$	Influx rate of chemical into cells	3.293 × 10^−5^	–	s^−1^
$k_{1}^{-}$	Efflux rate of chemical from cells	4.442 × 10^−6^	8.059 × 10^−8^	s^−1^
$k_{2}^{+}$	Association rate for riboswitch complex	6.962 × 10^−6^	–	(*μ*Ms)^−1^
$k_{2}^{-}$	Dissociation rate for riboswitch complex	2.239 × 10^−6^	–	s^−1^
*k*_3_	DsRed production rate from riboswitch complex	1.209 × 10^−4^	1.244 × 10^−4^	s^−1^
*k*_4_	DsRed destruction rate	7.342 × 10^−6^	7.342 × 10^−6^	s^−1^
*k*_5_	*De novo* DsRed synthesis in absence of riboswitch complex	1.299 × 10^−3^	1.299 × 10^−3^	*μ*M/s
*P*	Partition ratio of cytoplasm to media concentrations at equilibrium	–	7.413	–
*K*_*D*_	Dissociation constant between chemical and riboswitch in cytoplasm	–	0.3216	*μ*M
[r]_tot_	Total riboswitch concentration	3.481	3.512	*μ*M

Some parameter values were identified through curve-fitting to either the shown rate equations or Eq (3) or to DsRed reporter fluorescence data (see Materials & methods). Parameters fit to rate equations are referred to in this table as “computational” values, whereas those identified for Eq (3) are referred to as “mathematical” values. The cytosol-media partition ratio, *P*, was approximated through equation with an experimentally derived RDX octanol-water partition ratio value [20].

Finding an analytic model for the time-dependent, mean DsRed concentration, [*p*](*t*), allows for a greater understanding of how each internal process contributes to the average DsRed response, and would pave the way for rational designs of macroscale riboswitch sensor systems. Unfortunately, the coupled system of ordinary differential equations described in Table 1 has no general, closed-form solution. So we must yield to approximate methods to identify solutions that are of reasonable accuracy to provide mechanistic insight into the riboswitch system. One such method is the homotopy perturbation method [21], designed as a method to solve nonlinear differential equations. It is a perturbative approach that, in many cases, will converge sufficiently close to the exact solution upon calculation of only one or two terms in the series. We applied this method to the model of Table 1, but it failed to converge sufficiently close to the numerical solution by calculation of the 3^rd^ term in the series, which rendered it impractical for our purposes.

We resolved this problem by applying the method of time-scale decoupling to partition the model system into distinct sets of either “fast” or “slow” biochemical transformations. The advantage of this “quasi-steady-state hypothesis” approach [22] is that the assumed faster reactions are, to good approximation, in chemical equilibrium when compared against the states of the slower ones. This approximation is made in the spirit of the Briggs-Haldane steady-state approach [22], and applied here to simplify the mathematical description of DsRed protein production. When applied to the equations of Table 1, they yield

$$\begin{array}{c} \left[p\right](t)=\frac{k_{5}}{k_{4}}+({\left[p\right]}_{0}-\frac{k_{5}}{k_{4}})e^{-k_{4}t}+\int_{0}^{t}ds\;G(s)e^{-k_{4}(t-s)}, \end{array}$$ (1)

wherein [*p*]_0_ is the DsRed protein concentration measured at *t* = 0, and the function *G*(*s*) is defined as

$$\begin{array}{c} G(s)≔\frac{k_{3}{\left[r\right]}_{\text{tot}}}{1+\frac{K_{D}}{{\left[c\right]}_{\text{tot}}}\frac{1+P}{P}{\left(1-e^{(1+P)k_{1}^{-}s}\right)}^{-1}}. \end{array}$$ (2)

In these equations, *k*_5_/*k*_4_ is the steady-state DsRed concentration in absence of any chemical (i.e., *k*_3_ = 0); $P≔k_{1}^{+}/k_{1}^{-}>0$ is the partition ratio of chemical measured in cytoplasm to that in media and evaluated at long times (*t* → ∞); and $K_{D}≔k_{2}^{-}/k_{2}^{+}$ is the dissociation constant for chemical to riboswitch within cells.

Although the integrand of Eq (1) can be formally evaluated in terms of hypergeometric functions, this approach is rather complicated and obscures contributions from the relevant timescales to the DsRed dynamics. Alternatively, a reasonable and simple approximation to the full expression for [*p*](*t*), valid at longer times and larger concentrations, can be obtained by expanding Eq (2) in Taylor series to first order about *t* − *s* = 0. This choice of expansion point represents the largest contribution to the integrand of Eq (1), and should therefore encompass much of the contribution of *G*(*s*) to the integral of Eq (1). This leads to the following equation:

$$\begin{array}{c} \left[p\right](t)=\frac{k_{5}}{k_{4}}+({\left[p\right]}_{0}-\frac{k_{5}}{k_{4}})e^{-k_{4}t}\\ +[\frac{\left(1-e^{-k_{4}t})(1-e^{-(1+P)k_{1}^{-}t}\right)}{1+\frac{K_{D}}{{\left[c\right]}_{\text{tot}}}\frac{1+P}{P}-e^{-(1+P)k_{1}^{-}t}}-\frac{\frac{k_{1}^{-}}{k_{4}}\frac{K_{D}}{{\left[c\right]}_{\text{tot}}}\frac{{\left(1+P\right)}^{2}}{P}(1-(1+k_{4}t)e^{-k_{4}t})e^{-(1+P)k_{1}^{-}t}}{{\left(1+\frac{K_{D}}{{\left[c\right]}_{\text{tot}}}\frac{1+P}{P}-e^{-(1+P)k_{1}^{-}t}\right)}^{2}}]\frac{k_{3}}{k_{4}}{\left[r\right]}_{\text{tot}} \end{array}$$ (3)

As illustrated in S2 Fig, Eq (3) is very close to results from the computational model of Table 1: >84% and >96% agreement at, respectively, *μ*M and mM concentrations, for all but the shortest timescales. It also predicts accurate steady-state values:

$$\begin{array}{c} \lim_{t\to \infty }\left[p\right](t)=\frac{k_{5}}{k_{4}}+(\frac{{\left[c\right]}_{\text{tot}}}{K_{D}\frac{1+P}{P}+{\left[c\right]}_{\text{tot}}})\frac{k_{3}}{k_{4}}{\left[r\right]}_{\text{tot}}. \end{array}$$ (4)

As expected, the model predicts steady-state DsRed concentrations that exhibit sigmoid concentration-response as a function of the chemical dose in media, [*c*]_tot_.

### Model parameterization

Aptamer selection is achieved by screening an ensemble of aptamers with varying nucleic acid sequences for their binding affinity to target chemical [23]. Although our model was developed using data acquired for hexahydro-1,3,5-trinitro-1,3,5-triazine (RDX), it could similarly describe the sensor response to other chemicals. If chemical transport across lipid membranes and binding with the riboswitch can be modeled with first-order chemical kinetics, then our model is general enough that it could be applied to any number of water-soluble substances. Although we produced a number of aptamer clones that exhibited similar affinity to RDX as measured through binding assays [18], we fit our models to data acquired from the clone with the highest binding affinity (i.e., Sequence 11 as reported in [18]). But, in principle, we could reparameterize our models to any other such clone.

We can expect that values for our models’ parameters will generally be dependent on the specifics of the aptamer-target interaction, strength of the riboswitch regulation, and the average chemical influx and efflux rates into and out the cytosol of the *E. coli* culture. If the culture has not been exposed to chemical, then any subsequently measured DsRed response can be attributed to basal translational activity for DsRed within the cells. If we apply the condition [*c*]_tot_ = 0 to Eq (3), then we find that clone-specific control data can be used to identify the values for *k*_4_, *k*_5_, and [*p*]_0_ by using a curve-fitting methodology which minimizes an unweighted least-squares objective functional.

Curve-fitting is carried out by applying Eq (3) to fluorescence measurements, which we express as DsRed protein concentrations using a standard curve that empirically links DsRed concentrations in media (S1 Fig, abscissa) with an associated fluorescence response (S1 Fig, ordinate). All identified parameter values are collected into Table 1.

A value for the chemical-aptamer binding affinity (= 1/*K*_*D*_) can be found by using a cell-free kinetic assay from which the riboswitch complex yield can be modeled with the reaction mechanism: $c+r\overset{k_{2}^{+}}{\underset{k_{2}^{-}}{⇌}}r^{*}$. In this cell free assay, if we assume that riboswitch is not appreciably degraded, destroyed, or otherwise removed from the system, so that the total concentration is conserved, then [*r*](0) = [*r*]_0_. If we apply this condition in addition to an assumption that binding equilibrium is rapidly achieved, *d*[r*]/*dt* = 0, then we find:

$$\begin{array}{c} \left[r^{*}\right]=\frac{\left[c\right]}{K_{D}+\left[c\right]}{\left[r\right]}_{0}. \end{array}$$ (5)

Although *K*_*D*_ is formally the ratio of disassociation to association rate constants, a curve-fitting methodology that operates on logarithmically scaled RDX concentration data can be used to identify its value directly from the response of a binding assay (see S3 Fig): *K*_*D*_ = 0.3216[0.008968, 11.53] (95% confidence intervals in brackets).

**Fig 2:**
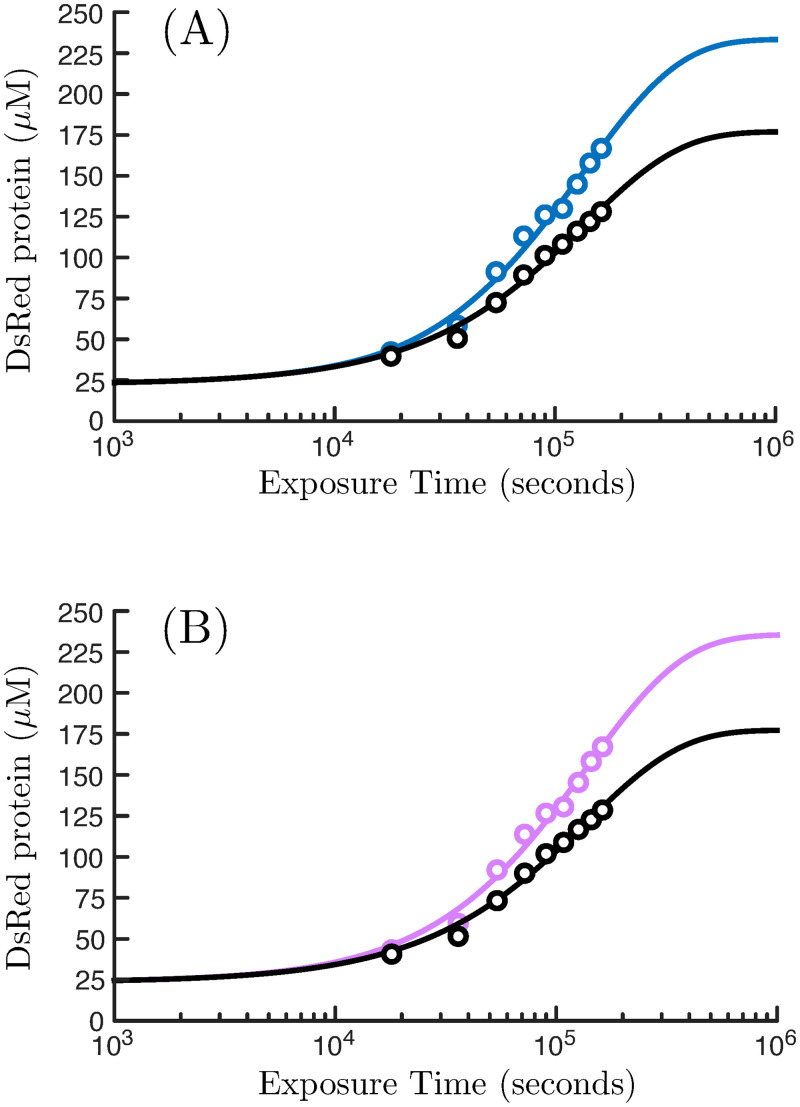
Time series data for the riboswitch system generated from aptamer sequence 11, as described in Eberly *et al.* [18]. Data represents both a control response (black circles) in addition to measurements from a 45 *μ*M exposure of RDX (blue circles). In this experiment, RDX concentrations are dissolved at time *t* = 0 in media populated with *E. coli* bacterium bearing the DsRed riboswitch reporter system. DsRed concentrations are found by transforming fluorescence data with a measured standard curve (S1 Fig). Panel (A) depicts these data fit to the ordinary differential equation (ODE) model shown in Table 1, while panel (B) shows these data alternatively fit to Eq (3). All fits were carried out by minimizing the value of a standard least-squares objective functional.

The long-time limit for the ratio of chemical concentration in cells to media is given by the equilibrium partition ratio, *P*, and can be reasonably estimated by the octanol-water partition ratio [24]: *P* = 7.413 [20]. The remaining undetermined parameter values of Eq (3) are the efflux rate constant, $k_{1}^{-}$, and total riboswitch concentration, [*r*]_tot_. These values were determined by fitting Eq (3) to the 45 *μ*M RDX exposure data (black circles, Fig 2).

## Results and discussion

### Fidelity of the model to the riboswitch sensing system

We evaluate the performance of Eq (3), given parameter values identified for the ODE model of Table 1 by curve-fitting the time-series data (Fig 2A), which permits a direct comparison between models and helps to establish the validity of many of the simplifying assumptions implicit in Eq (3). Specifically, if we put the “computational” parameter values listed in Table 1 into Eq (3), we generally find that the DsRed protein concentrations of both models closely agree, with the best agreement for long times and high exposure concentrations. Although model agreement varies with the RDX exposure (see S2 Fig), it is bounded between approximately 84% at the *μ*M level of exposure to 96% at the *μ*M level of exposure for incubation times between approximately 10^2^ and 10^7^ seconds.

We find that if DsRed measurements are made on bacteria only briefly incubated in exposed media, then Eq (3) is a poor substitute for the response of the full ODE model. (This result does not hold if the mathematical model is independently fit to the experimental data, as described below.) However, a riboswitch sensor that involves environmental placement and continuous sampling will likely experience time scales long enough that steady-state conditions can be reasonably assumed. In such circumstances, we can expect that Eq (3) will provide a reasonable model from which to interpret the riboswitch response data.

However, both models fit the time-series data quite well, and, as expected, make identical predictions of the steady-state response. Fig 2B shows the fidelity of Eq (3) if fit independently to time-series experimental data acquired for the aptamer clone Sequence 11 employed by Eberly *et al.* [18], for both control (*R*^2^ = 0.9903) and a 45 *μ*M RDX exposure (*R*^2^ = 0.9841). Both models capture dynamics of the DsRed response equally well, despite the moderate simplifying approximations that led to Eq (3).

### Linking exposure time to detection sensitivity

**Fig 3:**
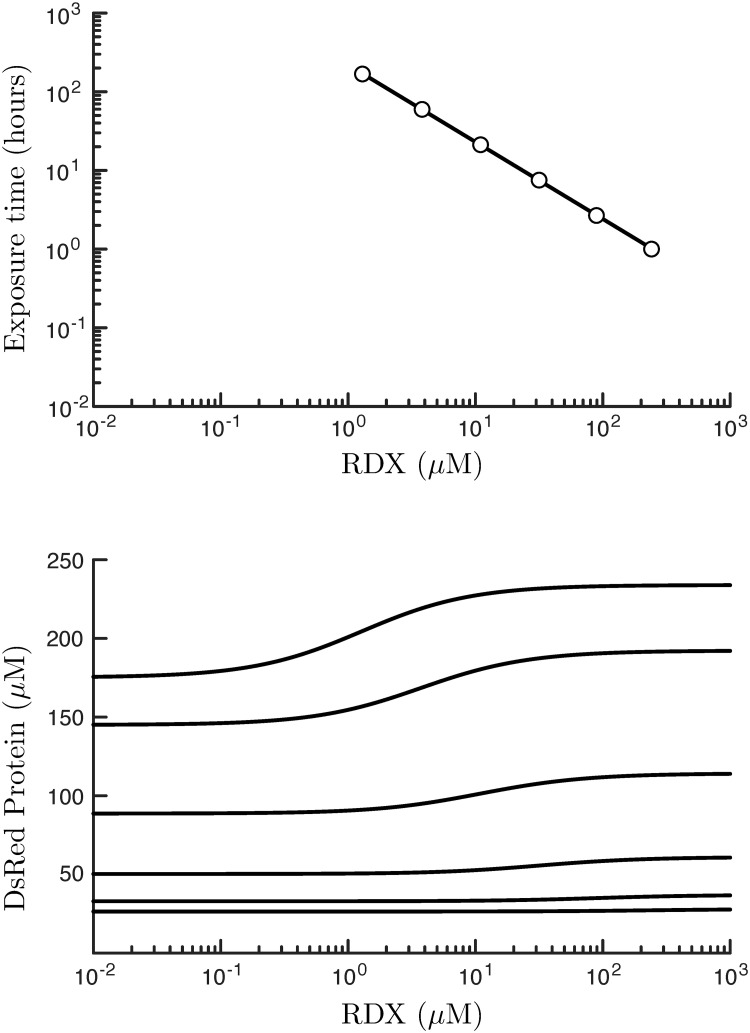
Relationship between the elapsed time of an RDX dose dissolved in media populated with *E. coli* bacteria, termed the exposure time, and the RDX concentrations associated with a half-maximal DsRed response (top panel, circles). Response-response curves that link the magnitude of an RDX exposure with an associated DsRed response are calculated using Eq (3) and shown for various exposure times (bottom panel, solid black lines).

Exposure-response predictions of Eq (3), as fit to the experimental data, are illustrated in Fig 3 (bottom panel), and calculated from RDX exposures ranging from 10 nM to 1 mM after a simulated exposure time of *t* = 1 to 168 hours. Of note is that, while the magnitude of the DsRed response increases with exposure time, making the overall response much more distinguishable from fluctuations in the surrounding environment, the difference between the initial and final responses, as measured over the whole of the exposure range, also increases with the exposure time. This relationship can inform sensor design, because the exposure time is an experimentally controllable parameter that can be chosen so as to exceed DsRed fluorescence detection thresholds.

We quantify the sensor sensitivity of the riboswitch system, as the total RDX concentration that elicits half of the maximal DsRed response, [*c*_50_], and can be found by solving the equation:

$$\begin{array}{c} \left[p\right](t;[c_{\text{50}}])=\frac{1}{2}(\left[p\right](t;0)+\lim_{{\left[c\right]}_{\text{tot}}\to \infty }\left[p\right](t;{\left[c\right]}_{\text{tot}})). \end{array}$$

Fig 3 illustrates how this sensor sensitivity varies with exposure time (top panel, circles). We find that [*c*_50_] decreases with longer RDX exposure times, meaning that the riboswitch system is able to register substantial fluorescence for even relatively low RDX exposures, but at the expense of a longer exposure time. We can fit a curve to this trend, which is found to follow a power-law: [*c*_50_] = (*τ*/*t*)^*γ*^ *μ*M. The fitted parameters, with 95% confidence intervals, are found to be *τ* = 219 [216, 224] hours, and *γ* = 1.0225 [1.0163, 1.0278]. Therefore, to very good approximation, the sensor sensitivity and exposure time are inversely proportional:

$$\begin{array}{c} \left[c_{\text{50}}\right]\approx \frac{219}{t}\;\mu \text{M}, \end{array}$$ (6)

for *t* in units of hours. As can be seen from Fig 3 (top panel), this equation implies that every 10-fold increase in the detection sensitivity requires an approximately 10 additional hours of exposure. Thus, rapid measurements need to be constrained by the lower detection limit of the fluorescence imager, because at these times the bacterial DsRed response is much less pronounced, and therefore weaker and harder to detect. Finally, it’s worth noting that this relationship does not hold indefinitely, because eventually, an idealized system will reach a steady state at an exposure time-scale on the order of $t∼\text{max}\{1/k_{1}^{-},1/k_{4}\}$. After this time, we can expect no further benefit from longer exposure times, because the riboswitch response should be well modeled by the time-independent Eq (4).

## Conclusion

Mathematical modeling of the riboswitch response indicated, somewhat counterintuitively, that basal DsRed expression in no-exposure conditions may significantly contribute to the overall magnitude of DsRed expression levels, which generally increases over moderate exposure times. However, correlated with this increase is both a larger difference in the response between the simulated lowest and highest RDX exposures, and an increase in the detection sensitivity of the riboswitch system with longer exposure times (see Eq (6)).

At short exposure times, RDX detection is constrained not only by high levels of extrinsic noise in the number of detected DsRed proteins (population heterogeneity), but also by the intrinsic noise inherent to the biochemistry (chemical influx/efflux rates, DsRed translation rates). As the number of DsRed proteins rise with longer exposure times, we can expect smaller fluctuations, and, therefore, a greater consistency in the DsRed response. This follows from the coefficient of variation in the number of proteins generated by a simple model of cellular protein abundance, in which a fixed probability for both protein creation and destruction of individual proteins is assumed (see, e.g., the model of ref. [25]); the resulting probability distribution is given by the Poisson distribution, which exhibits these properties. Additionally, direct simulation of the chemical master equation also generates this distribution [26], and for similar reasons. This is important, because the variance of a Poisson distribution is equal in magnitude to its mean, which results in a coefficient of variation that varies inversely with the square root of the mean number of molecules. Therefore, if protein production outweighs destruction, leading to an increase in the average number of proteins, then the spread of the distribution will decrease and becomes sharper, making our mean-field approach more relevant for riboswitch sensor system. Given the stability of the DsREd Express2 protein (>48 hours) [27], it is anticipated that that this will be the case.

With longer exposure times, overall DsRed fluorescence tends to increase (e.g., Fig 3), making these near-equilibrium conditions more capable of eliciting the effects of lower RDX exposure concentration, because the difference between the low and high RDX concentration response also grows with time. Practically, this empirical relationship can be used to deduce the minimum exposure time required to meet a minimum threshold for detection, which will, in principle, vary with fidelity of the fluorescent detector.

Finally, our models have been developed to best describe a reporter response that is averaged over bacterial populations, which have been assumed to persist in the exponential growth phase. However, such growth is limited by, among other resources, the availability of a carbon source. Realistically, *E. coli* remain in the exponential growth phase for <1 day, depending on the substrate concentration of the prepared media. Therefore, growth rates (or lack thereof) would be problematic for measuring and inferring reliable DsRed reporter response, as growth and division manifests in the model via protein dilution. This fact remains a major challenge for environmental detection via microorganisms, especially given that our results suggest that longer exposure times are required for detection of more environmentally relevant chemical concentrations. Therefore, complex soil and water matrices encountered in the environment may pose additional challenges to reliable detection of RDX and other chemicals, which could ultimately limit the environmental applicability of this biosensor to surface or ground water sources.

## Supporting information

S1 Fig DsRed standard curve. Standard curve measured between DsRed protein concentration and fluorescence readout of a BioTek Synergy HT plate reader. Data were fit to a linear model, [RFU] = slope × [DsRed], that quantitatively links DsRed protein concentration, [DsRed], to a measured fluorescent response expressed in relative fluorescence units, [RFU]. In our curve fitting protocol for the DsRed standard curve, we first logarithmically scaled the DsRed protein and associated fluorescent response data, because the DsRed protein concentration and fluorescence data both span multiple orders of magnitude in value. Taking the logarithm of these data puts lower values on similar scales as the higher values, which avoids a situation in which changes to the larger values in the least squares objective functional wash out a change in the smaller values. This would otherwise erroneously produce a bias toward the higher-valued elements of the dataset. The curve-fitted result is slope = 107.31[50.895, 226.24] (95% confidence intervals in brackets).
(TIF)

S2 Fig Validity of mathematical approximations. Minimum agreement across all simulated elapsed times between the ODE based model (Table 1) and Eq (3) of the main text, as plotted against a range of total RDX concentration values. The approximate model, Eq (3), is quantitatively closer to predictions of the chemical kinetics model, Table 1, for larger total RDX concentrations.
(TIF)

S3 Fig Aptamer clone binding assay. Cell free binding assay used to estimate binding affinity of RDX to aptamer clone. These data correspond to the aptamer clone associated with the highest binding affinity to RDX. Refer to Section IIC of the main text for further information. A least squares objective functional was minimized using log-transformed data to result in *K*_*D*_ = 0.3216[0.008968, 11.53] (95% confidence intervals in brackets).
(TIF)
