# Supplementary figures and images for: Modeling a synthetic aptamer-based riboswitch biosensor sensitive to low hexahydro-1,3,5-trinitro-1,3,5-triazine (RDX) concentrations
Source: PLoS One. 2020 Nov 30;15(11):e0241664. doi: 10.1371/journal.pone.0241664 (PMC7703952; doi:10.1371/journal.pone.0241664)

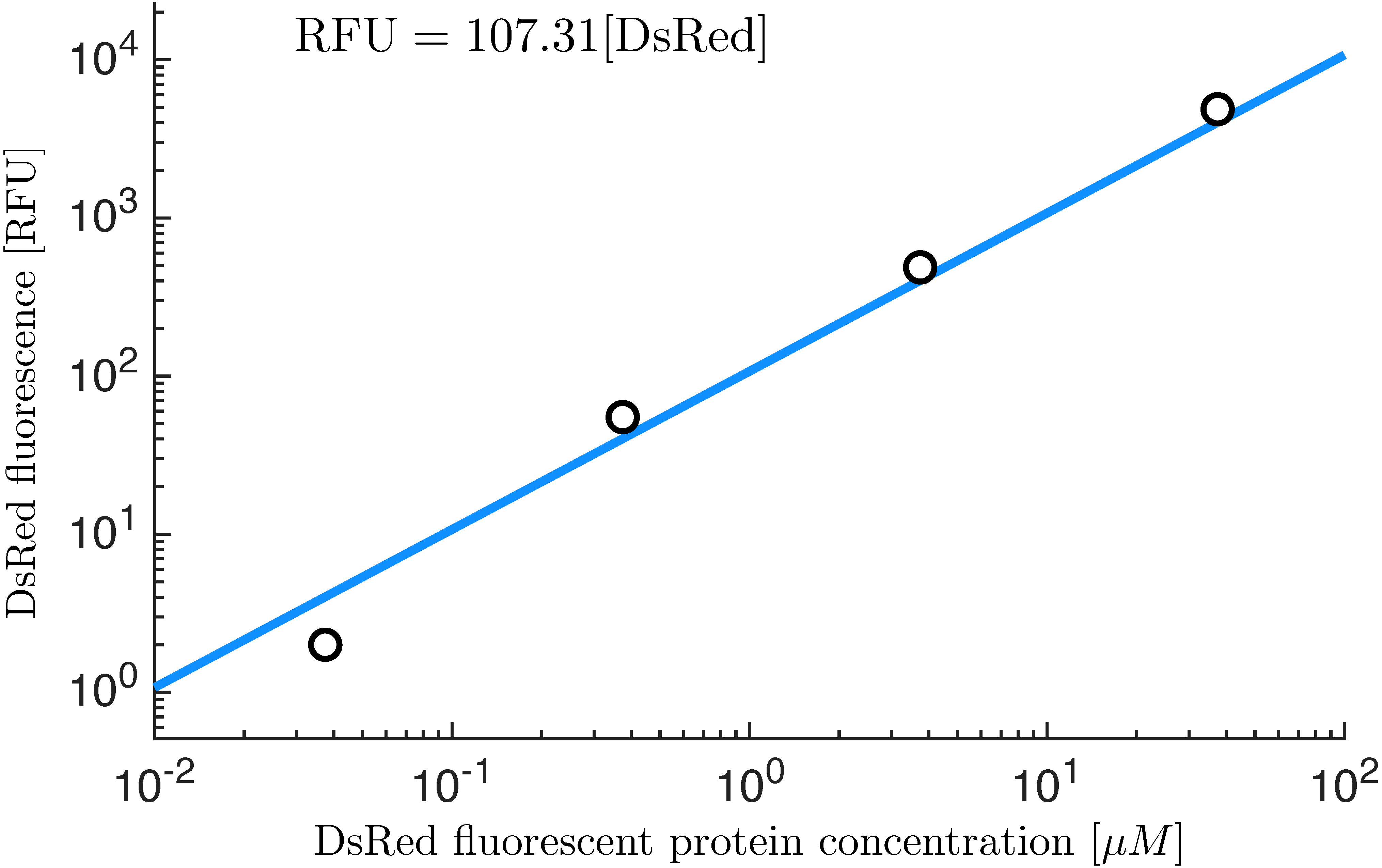

Supplement: S1 Fig — Standard curve measured between DsRed protein concentration and fluorescence readout of a BioTek Synergy HT plate reader. Data were fit to a linear model, [RFU] = slope × [DsRed], that quantitatively links DsRed protein concentration, [DsRed], to a measured fluorescent response expressed in relative fluorescence units, [RFU]. In our curve fitting protocol for the DsRed standard curve, we first logarithmically scaled the DsRed protein and associated fluorescent response data, because the DsRed protein concentration and fluorescence data both span multiple orders of magnitude in value. Taking the logarithm of these data puts lower values on similar scales as the higher values, which avoids a situation in which changes to the larger values in the least squares objective functional wash out a change in the smaller values. This would otherwise erroneously produce a bias toward the higher-valued elements of the dataset. The curve-fitted result is slope = 107.31[50.895, 226.24] (95% confidence intervals in brackets). (TIF) [file pone.0241664.s001.tif]

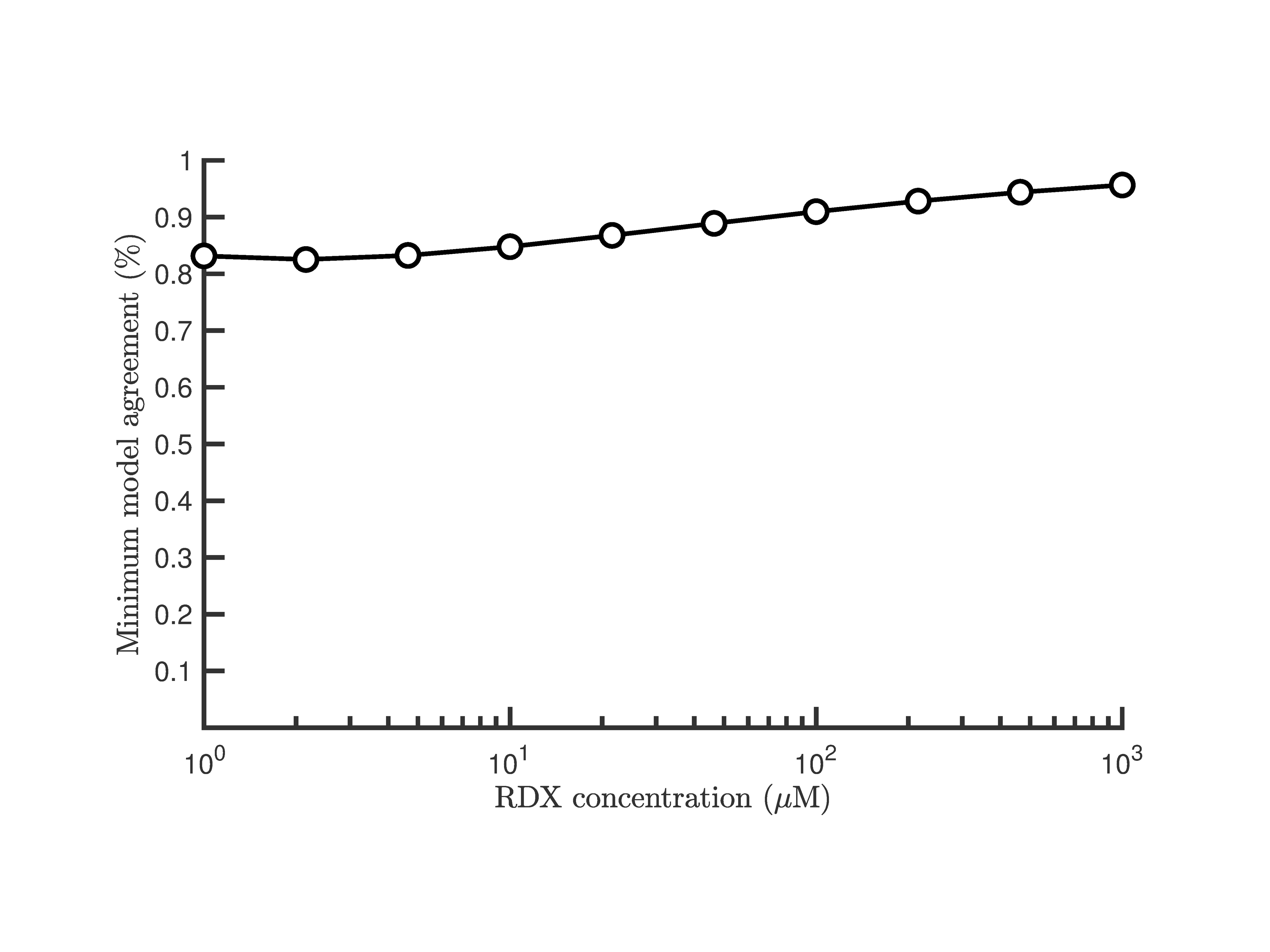

Supplement: S2 Fig — Minimum agreement across all simulated elapsed times between the ODE based model (Table 1) and Eq (3) of the main text, as plotted against a range of total RDX concentration values. The approximate model, Eq (3), is quantitatively closer to predictions of the chemical kinetics model, Table 1, for larger total RDX concentrations. (TIF) [file pone.0241664.s002.tif]

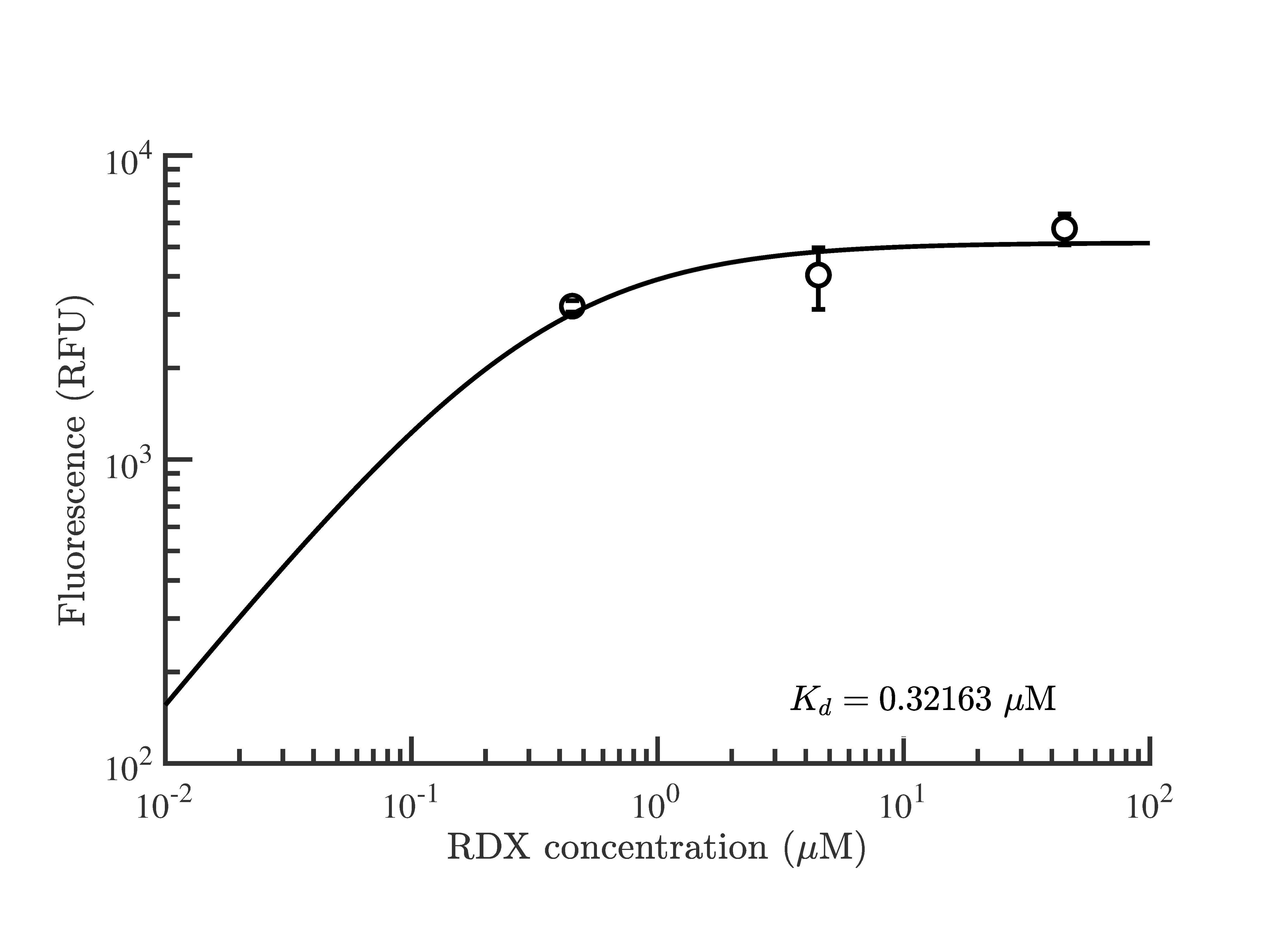

Supplement: S3 Fig — Cell free binding assay used to estimate binding affinity of RDX to aptamer clone. These data correspond to the aptamer clone associated with the highest binding affinity to RDX. Refer to Section IIC of the main text for further information. A least squares objective functional was minimized using log-transformed data to result in KD = 0.3216[0.008968, 11.53] (95% confidence intervals in brackets). (TIF) [file pone.0241664.s003.tif]
